# Menstrual and reproductive factors and risk of breast cancer: A case-control study in the Fez region, Morocco

**DOI:** 10.1371/journal.pone.0191333

**Published:** 2018-01-16

**Authors:** Mohamed Khalis, Barbara Charbotel, Véronique Chajès, Sabina Rinaldi, Aurélie Moskal, Carine Biessy, Laure Dossus, Inge Huybrechts, Emmanuel Fort, Nawfel Mellas, Samira Elfakir, Hafida Charaka, Chakib Nejjari, Isabelle Romieu, Karima El Rhazi

**Affiliations:** 1 Department of Epidemiology, Faculty of Medicine and Pharmacy, Fez, Morocco; 2 University Claude Bernard Lyon1, Ifsttar, UMRESTTE, UMR T_9405, Lyon, France; 3 Sidi Mohamed Ben Abdellah University, Fez, Morocco; 4 Section of Nutrition and Metabolism, International Agency for Research on Cancer, Lyon, France; 5 Department of Oncology, Hassan II University Hospital of Fez, Fez, Morocco; 6 Mohammed VI University of Health Sciences, Casablanca, Morocco; Baylor College of Medicine, UNITED STATES

## Abstract

**Background:**

Breast cancer is the most common cancer in women worldwide. In the Moroccan context, the role of well-known reproductive factors in breast cancer remains poorly documented. The aim of this study was to explore the relationship between menstrual and reproductive factors and breast cancer risk in Moroccan women in the Fez region.

**Methods:**

A case–control study was conducted at the Hassan II University Hospital of Fez between January 2014 and April 2015. A total of 237 cases of breast cancer and 237 age-matched controls were included. Information on sociodemographic characteristics, menstrual and reproductive history, family history of breast cancer, and lifestyle factors was obtained through a structured questionnaire. Conditional logistic regression models were used to estimate odds ratios and 95% confidence intervals for breast cancer by menstrual and reproductive factors adjusted for potential confounders.

**Results:**

Early menarche (OR = 1.60, 95% CI: 1.08–2.38) and nulliparity (OR = 3.77, 95% CI: 1.98–7.30) were significantly related to an increased risk of breast cancer, whereas an early age at first full-term pregnancy was associated with a decreased risk of breast cancer (OR = 0.41, 95% CI: 0.25–0.65).

**Conclusion:**

The results of this study confirm the role of established reproductive factors for breast cancer in Moroccan women. It identified some susceptible groups at high risk of breast cancer. Preventive interventions and screening should focus on these groups as a priority. These results should be confirmed in a larger, multicenter study.

## Introduction

Breast cancer is the most commonly diagnosed cancer in women worldwide [1]. The etiology of female breast cancer is multifactorial, and includes reproductive, genetic, lifestyle, and environmental factors [2–5].

In North Africa, as in many regions that are either developing or in epidemiological transition, breast cancer incidence rates have clearly risen [6]. In Morocco, breast cancer remains the most common cancer in women, constituting about 35.8% of all new cancer diagnoses in women [7]. According to the most recent report of the cancer registry in Casablanca, the age-standardized incidence rate of breast cancer increased from 35.0 to 49.5 per 100 000 women between 2004 and 2012, showing an annual increase of 3.18%. The most widely proposed explanations for this increase are changes in reproductive behaviors (smaller number of children, shorter duration of breastfeeding, higher age at first pregnancy), and in lifestyle and dietary habits (higher obesity rates) among Moroccan women in the past three decades [8–10].

Numerous epidemiological studies performed throughout the world have confirmed the role of many reproductive factors, such as age at menarche, age at first pregnancy, age at menopause, parity, and breastfeeding, in the etiology of breast cancer [2,11,12].

However, evidence suggests that international variation in the burden of breast cancer reflects differences in the patterns of risk factors [13]. In the Moroccan context, the role of well-known reproductive factors in breast cancer remains poorly documented, and it is probable that unidentified exposures specific to Moroccan women may play an important role.

The aim of this case–control study was to explore the relationship between menstrual and reproductive factors and breast cancer risk in Moroccan women in the Fez region. To the best of our knowledge, this is one of the first epidemiological studies on risk factors for breast cancer in Morocco.

## Methods

### Study design and setting

The Fez Breast Cancer Study is a case–control study, conducted at the Hassan II University Hospital of Fez, ranked as one of the most important medical centers in Morocco, covering more than 3 million people in the Fez region.

In this study, a total of 474 women (237 cases and 237 age-matched controls) were recruited between January 2014 and April 2015.

### Study subjects

Cases were patients recently diagnosed with histologically confirmed breast cancer (all consecutive cases), admitted during the study period to the Medical Oncology Center at the University Hospital of Fez, which is a referral center for breast cancer in the region.

Controls were healthy women with no history of cancer, who accompanied patients to the consultations department at the University Hospital of Fez. This department provides health consultations to patients for various medical and surgical specialties. Women who accompanied patients with any type of cancer were excluded from this study. Control subjects were individually matched to cases by age (age at cancer diagnosis ± 3 years), and they were recruited at approximately the same time as the recruitment of cases.

Because of the difficulty of recruiting some controls aged 60 years or older among the women accompanying patients to the hospital, women randomly selected at the consultations department were asked whether they could provide the telephone numbers and addresses of other, older women, who could potentially be recruited. Women meeting the age requirement were called by telephone, checked for inclusion criteria, and invited to participate in this study. The socioeconomic characteristics such as income and education level of controls recruited with this method are approximately the same as those of other controls (data not shown).

The participation rate in this study was 97.1% (237/244) for cases and 92.5% (237/256) for controls.

### Ethical considerations

This study was approved by the Ethics Committee of the Hassan II University Hospital of Fez. Participation in this study was strictly voluntary, and all subjects were informed about the right to withdraw at any time without giving an explanation. All information collected from participants was kept confidential. The Fez Breast Cancer Study was conducted according to the guidelines of the Declaration of Helsinki. Written informed consent to participate in the study was obtained from all study participants before the interviews were conducted.

### Data collection and measurements

Data were collected through face-to-face interviews by six trained interviewers. The pre-tested structured questionnaire included information on sociodemographic characteristics (e.g., age, educational level, marital status, area of residence, occupation, monthly household income), menstrual and reproductive history (e.g., age at menarche, regularity of menstrual cycle, age at menopause, parity, age at first full-term pregnancy, history of abortions, history of miscarriages, use of oral contraceptives, breastfeeding, postmenopausal hormone use), family history of breast cancer in first- and second-degree relatives, alcohol consumption, smoking, and passive smoking (the inhalation of tobacco smoke from people who are smoking nearby in the home or at work).

Current weight and height were measured by the interviewers according to the recommendations of Lohman et al. (1988) [14]. Body mass index (BMI) was calculated as the weight in kilograms divided by the square of the height in meters. BMI was classified using cut-off points recommended by WHO [15]. The categories underweight (<18.5 kg/m^2^) and normal weight (18.5–24.9 kg/m^2^) were grouped into one category (<25 kg/m^2^), due to the low number of women in these categories. Information on physical activity was obtained using a Global Physical Activity Questionnaire (GPAQ2), which includes estimates of physical activity in three domains (activity at work, activity travelling to and from places, recreational activities) as well as sedentary behavior [16]. Definitions of these three domains are given in (WHO 2016) [16]. The Metabolic Equivalent (MET)-minutes per week were calculated based on the published GPAQ Analysis Guide [16]. The intensity of physical activity was classified into three categories: light intensity (<600 MET-minutes per week), moderate intensity (600–3000 MET-minutes per week), and vigorous intensity (≥3000 MET-minutes per week).

To determine menopausal status at recruitment, women were considered to be premenopausal if they reported regular menstrual cycles over the previous 12 months and postmenopausal in case of absence of menstruation in the last 12 months. Women who had missing data on menopausal status were considered to be premenopausal if they were younger than 45 years, and postmenopausal if they were older than 54 years, because in a previous study conducted in Moroccan women, the median age at menopause was estimated to be 50.25 years [17]. Women in the age range 45–54 years were assigned to an unknown menopausal status.

### Statistical analysis

Frequencies (percentages) for qualitative variables and mean values (± standard deviation) for continuous variables were calculated. Conditional logistic regression models were used to identify the reproductive factors associated with breast cancer. Multivariate adjusted odds ratios (ORs) and corresponding 95% confidence intervals (CIs) were estimated, adjusting for area of residence (urban, rural), monthly household income (≤2000, >2000 Moroccan Dirham), age at menarche (continuous), menopausal status and age at menopause combined (premenopausal, postmenopausal <46.5 years, postmenopausal ≥46.5 years, missing), parity (parous, nulliparous), age at first full-term pregnancy (nulliparous, <20 years, ≥20 years), family history of breast cancer (yes/no), and BMI (<25, 25–29, ≥30 kg/m^2^). None of other potential confounders listed in Table 1 changed our risk estimates by 10% or more. Median values in controls were used as cut-off points for age at menarche, age at menopause, age at first full-term pregnancy, and breastfeeding per child. BMI information was missing for three controls, and these missing values were replaced with the median value in controls. Data analysis was performed using Stata/IC 14.1 software.

**Table 1:** Baseline characteristics of case and control subjects [number (percentage) or mean ± standard deviation].

Characteristics	Cases	Controls
	(n = 237)	(n = 237)
**Age at recruitment** (years)		
<40	52 (21.9)	48 (20.3)
40–49	72 (30.4)	80 (33.8)
50–59	70 (29.5)	68 (28.7)
≥60	43 (18.1)	41 (17.3)
**Area of residence**		
Urban	161 (67.9)	183 (77.2)
Rural	76 (32.1)	54 (22.8)
**Marital status**		
Single	34 (14.3)	17 (7.2)
Married	153 (64.6)	173 (73.0)
Divorced	23 (9.7)	16 (6.8)
Widowed	27 (11.4)	31 (13.1)
**Educational level**		
Illiterate	151 (63.7)	153 (64.5)
Elementary school	44 (18.6)	38 (16.0)
Secondary school	30 (12.6)	30 (12.6)
High school	10 (4.2)	14 (5.9)
Unknown	2 (0.8)	2 (0.8)
**Monthly household income** (MAD)		
>2000	140 (59.1)	173 (73.0)
≤2000	97 (40.9)	64 (27.0)
**Occupation**		
Housewife	214 (90.3)	211 (89.0)
Employed	23 (9.7)	26 (11.0)
**Menopausal status**		
Premenopausal	100 (42.1)	124 (52.3)
Postmenopausal	129 (54.4)	106 (44.7)
Unknown	8 (3.3)	7 (2.9)
**Parity**		
Parous	181 (76.4)	201 (84.8)
Nulliparous	56 (23.6)	36 (15.2)
**Family history of breast cancer**		
No	198 (83.5)	224 (94.5)
Yes	39 (16.5)	13 (5.5)
**Passive smoking**		
No	131 (55.3)	139 (58.6)
Yes	106 (44.7)	98 (41.4)
**Intensity of physical activity**		
Light and moderate intensity	158 (66.7)	147 (62.0)
Vigorous intensity	79 (33.3)	90 (38.0)
**Body mass index** (kg/m^2^)		
<25	40 (16.9)	61 (25.7)
25–29	86 (36.3)	81 (34.2)
≥30	111 (46.8)	95 (40.1)
**Age at menarche** (years)	13.7 ± 1.7	14.0 ± 1.8
**Age at menopause** (years)^a^	47.7 ± 5.9	47.2 ± 5.4
**Age at first full-term pregnancy** (years)	22.2 ± 5.4	20.9 ± 4.9
**Interval between age at menarche and age at first full-term pregnancy** (years)^b^	8.4 ± 5.6	6.8 ± 5.0
**Interval between age at menarche and age at menopause** (years)^a^	33.8 ± 6.5	33.0 ± 5.7
**Breastfeeding per child** (months)^c^	12.0 ± 7.3	12.2 ± 7.1

MAD: Moroccan Dirham.

^a^Among postmenopausal women;

^b^Among parous women;

^c^Among breast-feeding women.

## Results

Table 1 presents the baseline characteristics of the subjects by case–control status. The mean age of the study population (cases and controls) was 48.6 years. Compared with control subjects, cases were more likely to be postmenopausal, nulliparous, younger at menarche, and older at first pregnancy, to live in a rural area, and to have a higher BMI, a family history of breast cancer, a lower monthly household income, and a longer interval between age at menarche and age at first full-term pregnancy. Cases and control subjects did not show differences in marital status, educational level, occupation, passive smoking, intensity of physical activity, age at menopause, breastfeeding per child, or interval between age at menarche and age at menopause.

Table 2 shows crude and adjusted ORs and 95% CIs for breast cancer by menstrual and reproductive factors. After adjustment for potential confounders, women who reached menarche at age ≤13 years had a significantly higher risk of breast cancer, compared with women who reached menarche after age 13 years (OR = 1.60, 95% CI: 1.08–2.38). Nulliparous women had a significantly higher risk of breast cancer compared with parous women (OR = 3.77, 95% CI: 1.98–7.30). Women who were younger than 20 years at their first full-term pregnancy had a significantly lower risk of developing breast cancer, compared with women who were older than 20 years at their first full-term pregnancy (OR = 0.41, 95% CI: 0.25–0.65).

**Table 2:** Association between menstrual and reproductive factors and breast cancer risk.

Characteristics	Cases /controls	Crude OR (95% CI)	Adjusted OR^a^ (95% CI)
**Age at menarche** (years)			
>13	118/145	Reference	Reference
≤13	119/92	1.59 (1.09–2.31)	1.60 (1.08–2.38)
**Regularity of menstrual cycle**			
Regular	222/223	Reference	Reference
Irregular	15/12	1.20 (0.51–2.77)	1.24 (0.54–2.85)
**Age at menopause** (years)^b^			
<46.5	51/46	Reference	Reference
≥46.5	62/46	1.72 (0.82–3.63)	1.26 (0.69–2.30)
**Parity**			
Parous	181/201	Reference	Reference
Nulliparous	56/36	1.85 (1.12–3.04)	3.77 (1.98–7.30)
**History of miscarriages**			
No	166/161	Reference	Reference
Yes	69/76	0.88 (0.61–1.28)	0.96 (0.61–1.51)
**History of abortions**			
No	208/216	Reference	Reference
Yes	27/21	1.32 (0.71–2.45)	1.59 (0.83–3.04)
**Age at first full-term pregnancy** (years)			
≥20	117/102	Reference	Reference
<20	63/99	0.56 (0.34–0.92)	0.41 (0.25–0.65)
**Breastfeeding per child** (months)^c^			
<11	86/93	Reference	Reference
≥11	86/94	0.90 (0.53–1.51)	1.0 (0.52–1.93)
**History of oral contraceptive use**			
No	89/92	Reference	Reference
Yes	148/145	1.05 (0.71–1.54)	1.11 (0.74–1.66)

OR: odds ratio; CI: confidence interval.

^a^Odds ratios adjusted for area of residence (urban, rural), monthly household income (≤2000, >2000 Moroccan Dirham), age at menarche (continuous), menopausal status and age at menopause combined (premenopausal, postmenopausal <46.5 years, postmenopausal ≥46.5 years, missing), parity (parous, nulliparous), age at first full-term pregnancy (nulliparous, <20 years, ≥20 years), family history of breast cancer (yes/no), and body mass index (<25, 25–29, ≥30 kg/m^2^);

^b^Among postmenopausal women;

^c^Among breastfeeding women.

Finally, there was no significant association with breast cancer risk for irregularity of menstrual cycle, age at menopause, history of miscarriages and abortions, breastfeeding per child, or history of oral contraceptive use.

## Discussion

The purpose of this study was to explore the relationship between menstrual and reproductive factors and breast cancer risk among Moroccan women in the Fez region.

The results from this case–control showed that early menarche (≤13 years) and nulliparity were significantly associated with an increased risk of breast cancer, whereas an early age at first full-term pregnancy (<20 years) was associated with a significantly decreased risk of breast cancer.

Consistent with findings from other epidemiological studies in Morocco [18] and other countries [19–22], we found a significant association between early age at menarche and an increased risk of breast cancer. A meta-analysis of 117 epidemiological studies confirmed that young age at menarche is associated with increased risk of breast cancer [23]. This meta-analysis showed that for every year of younger age at menarche, breast cancer risk increased by a factor of 1.05 (95% CI: 1.04–1.05). The biological explanation for this association is based on the early and prolonged exposure of the breast epithelium to estrogens produced during the period of activity of the ovaries [24].

In most studies in the literature, nulliparity was one of the strongest risk factors for breast cancer [21,25,26]. In line with these studies, we also found a positive association between nulliparity and breast cancer risk. Several mechanisms have been proposed to explain the potential protective effect of pregnancy on breast cancer, such as decreased levels of estrogen and progesterone, increased levels of sex hormone-binding globulin, and pregnancy-induced differentiation of breast tissue [27,28]. Further investigations are needed to explore the mechanisms underlying the positive association between nulliparity and breast cancer risk reported in our study.

In addition, a decrease in breast cancer risk with an increasing number of live births has been reported by many studies [29–32]. Clavel-Chapelon and Gerber indicated that each full-term pregnancy leads to a 3% reduction in the risk of breast cancer diagnosed early or in premenopausal women, while this reduction reaches 12% for cancers diagnosed in postmenopausal women [33].

In our study, an early age at first full-term pregnancy was associated with a reduced risk of breast cancer. Women who were younger than 20 years at first full-term pregnancy had a significant decreased risk of breast cancer compared with women who were older than 20 years. This finding may be explained by the fact that, at the first birth, the mammary epithelial cells, which have a high degree of terminal differentiation, are capable of metabolizing carcinogens and can repair DNA damage more efficiently [28]. Our result were in line with the findings of previously published studies in other populations [26,29,34,35].

There is evidence suggesting that breastfeeding may have a protective effect on breast cancer risk [36]. A meta-analysis including 50 302 women with breast cancer and 96 973 controls found that the relative risk of breast cancer decreased by 4.3% for every 12 months of breastfeeding [36]. Other studies also supported the inverse relationship between breastfeeding and breast cancer [25,37–39]. In our study, there is a lack of significant association between breastfeeding and breast cancer risk. This may be related to the prolonged breastfeeding practiced by both cases and controls in our study population and, more generally, in the region. The distinct exposure pattern of our population should be considered in the interpretation. In this study, we assessed the relationship between breastfeeding and breast cancer risk without considering hormone receptor status. Some studies have suggested that the effect of breastfeeding may differ by breast cancer subtypes [40–43]. Therefore, further studies are required to explore probable differences by tumor subtypes in our population.

A late age at menopause is a well-established risk factor for breast cancer [12]. Numerous studies [22,30,34,44] have shown a significant association between a later age at menopause and increased risk of breast cancer, while other studies, including ours, failed to report a significant association [19,45]. The higher risk of developing breast cancer in women with a late age at menopause may be explained by both longer duration of and higher level of exposure to estrogen and progesterone experienced by these women [12].

In agreement with results from another case–control study in the region [20], our study did not find a statistically significant association between oral contraceptive use and risk of breast cancer. However, some previous studies found that oral contraceptive use was associated with an increased risk of breast cancer [46,47]. Age at starting oral contraceptive use might be determinant in the association between oral contraceptive use and breast cancer risk; however, we did not have information on that variable, so we were unable to further assess its effect in our study.

The relationship between history of abortions and breast cancer risk is controversial [48]. In the current study, we found that history of abortions is not significantly associated with breast cancer risk. This result is in line with a meta-analysis of 53 epidemiological studies, including 83 000 women with breast cancer from 16 countries [49]. However, a recent meta-analysis conducted in China reported a positive association between breast cancer risk and induced abortion [50]. More details about abortion (number, age at abortion) may provide important information on the relationship between abortion and breast cancer risk.

Our study has some limitations. First, due to the retrospective nature of this study, most of our data were self-reported by women, and could be subject to recall bias. However, women were not aware of potential risk factors for breast cancer, and therefore measurement error is most likely to be random (non-differential misclassification). Another limitation is the relatively small number of cases and controls, which impaired stratified analysis by menopausal status, and factors with small effect on breast cancer risk may have been missed because of low statistical power. In addition, considering that cases were significantly more likely to live in a rural area and to have a lower monthly household income, compared with controls, the possibility of selection bias cannot be ruled out in this study.

The probable explanation is that controls were recruited from a consultations department located in an urban area, and those accompanying patients to this department are more likely to reside in an urban area and consequently their socioeconomic status may have been relatively high. The epidemiological transition and the rise of chronic diseases associated with urban lifestyles that are beginning to be seen among Moroccan women of lower socioeconomic status may be another possible explanation for this difference. It is therefore particularly important to explore the relationship between the socioeconomic level and breast cancer risk in the Moroccan context.

Moreover, in this study some variables were excluded from the analysis, due to the low number of women with certain habits in the region of the study (the frequency of postmenopausal hormone use was 1.68% in cases and 0.84% in controls, the frequency of smoking was 4.64% in cases and 0.42% in controls, and the frequency of alcohol consumption was 0.00% in cases and 0.42% in controls).

This study has several strengths, including an age-matched case–control design, a high participation rate in cases and controls, and histological confirmation of breast cancer. Moreover, this is the first epidemiological study to investigate the relationship between reproductive factors and risk of breast cancer in the Fez region.

Our study confirms the role of established reproductive factors for breast cancer in Moroccan women. Studies exploring changes in the pattern of breast cancer risk factors in Morocco [8,10] suggest that adoption of a Western lifestyle, birth-control policies, and changes in dietary habits might have resulted in earlier age at menarche, later age of marriage and first pregnancy, and a decline in fertility.

Finally, the results of this study identified some susceptible groups at high risk of breast cancer. Preventive interventions and screening should focus on these groups as a priority. These results should be confirmed in a larger, multicenter study to support the generalization of the results to all Moroccan women.

## Abbreviations

BMI: Body mass index
CI: confidence interval
GPAQ: Global Physical Activity Questionnaire
MET: Metabolic equivalent
OR: odds ratios
